# Placental Vulnerability to SARS-CoV-2: Viral Entry Pathways and Immune Activation

**DOI:** 10.3390/v18040426

**Published:** 2026-03-31

**Authors:** Madhumitha Natarajan, Bindu Jayashankar, Raghu Nataraj

**Affiliations:** 1 Division of Molecular Biology, School of Life Sciences, Jagadguru Shri Shivaratreeshwara (JSS) Academy of Higher Education and Research, Mysore 570015, Karnataka, India; 25plm001@jssuni.edu.in; 2 Department of Biotechnology, Sri Jayachamarajendra College of Engineering, Mysore 570006, Karnataka, India

**Keywords:** SARS-CoV-2, placenta, pregnancy, maternal–fetal interface, immune response, viral entry

## Abstract

Pregnancy represents a distinct immunological and physiological state that modifies maternal susceptibility to SARS-CoV-2 and influences the clinical and biological course of COVID-19. Accumulating evidence indicates that the interaction between viral entry determinants, gestation-specific immune modulation, placental endocrine–angiogenic pathways, and systemic inflammatory responses underlies the characteristic manifestations of SARS-CoV-2 infection during pregnancy. This review consolidates current understanding of SARS-CoV-2 viral structure, receptor biology, and the gestational regulation of key entry cofactors, including ACE2, TMPRSS2, NRP1, CTSL and FURIN, within reproductive and placental tissues. The review further integrates documented mechanisms of cytokine-mediated immune dysregulation, endothelial injury, thrombo-inflammation, and steroidogenic alteration observed in affected pregnancies, and examines their contribution to placental malperfusion, preeclampsia-like presentations, fetal growth abnormalities and preterm birth. Published molecular and computational studies characterising trophoblast antiviral defenses, receptor expression patterns, and structural determinants of Spike–ACE2 affinity are synthesised to contextualise the biological basis of placental susceptibility and the rarity of confirmed transplacental transmission. Current evidence on maternal clinical outcomes, fetal and neonatal consequences, vaccination efficacy, therapeutic considerations and contemporary management guidelines is also critically reviewed. By integrating molecular, immunological, pathological and clinical insights, this article provides a comprehensive framework for understanding the interaction between SARS-CoV-2 infection and pregnancy-specific physiology, with implications for risk assessment, preventive strategies and maternal–fetal care.

## 1. Introduction

The coronavirus disease 2019 (COVID-19) pandemic, caused by severe acute respiratory syndrome coronavirus 2 (SARS-CoV-2), continues to present substantial global health challenges, extending beyond classic respiratory pathology to influence multiple organ systems and vulnerable populations. Among these, pregnant individuals represent a uniquely susceptible group due to the dynamic interplay between gestational immune modulation, altered physiological states, and the distribution of viral entry receptors in reproductive tissues. Notably, although confirmed placental infection by SARS-CoV-2 remains uncommon, maternal infection during pregnancy has been strongly associated with increased risks of maternal morbidity and adverse perinatal outcomes, including preterm birth, pre-eclampsia, fetal growth abnormalities, and neonatal complications [1,2]. This apparent paradox—significant obstetric and neonatal morbidity despite infrequent detection of placental viral invasion—raises critical mechanistic questions regarding how SARS-CoV-2 perturbs pregnancy biology.

Physiological adaptations inherent to pregnancy—including cardiovascular, respiratory, endocrine, and immunological changes—modify both susceptibility to infection and the clinical trajectory of disease. For example, pregnancy is characterised by a shift toward a Th2-dominant immune environment, which may attenuate antiviral defence mechanisms, potentially hindering efficient viral clearance and predisposing to prolonged inflammatory responses [3]. Mechanical factors such as diaphragmatic elevation and reduced functional residual capacity further compromise pulmonary reserve, complicating maternal responses to respiratory viral infections [4].

SARS-CoV-2 primarily enters host cells through binding of its Spike glycoprotein to the angiotensin-converting enzyme 2 (ACE2) receptor, a process facilitated by proteolytic activation via transmembrane serine protease 2 (TMPRSS2) [4]. Both ACE2 and TMPRSS2 exhibit gestationally regulated expression in placental tissues, including syncytiotrophoblasts, cytotrophoblasts, extravillous trophoblasts, and decidual stromal cells [5]. This spatial and temporal distribution suggests potential windows of susceptibility for SARS-CoV-2 interaction with the placenta. However, confirmed placental infection remains uncommon and, when present, is typically focal. This may reflect receptor polarisation, low co-expression of entry cofactors, and the presence of robust trophoblast antiviral defences, particularly at term [6].

Despite the infrequent detection of viral RNA in placental tissues, SARS-CoV-2 infection is consistently associated with heightened immune activation at the maternal–fetal interface and generalized systemic inflammation, both of which may contribute to adverse pregnancy outcomes independent of local viral replication [7,8]. Such immunological alterations include activation of natural killer (NK) cells, T-cell dysregulation, and induction of interferon-stimulated genes, each with significant implications for trophoblast differentiation, vascular homeostasis, and fetal development [9].

Clinically, maternal SARS-CoV-2 infection has been linked to increased risks of obstetric complications and neonatal morbidity, including preterm labour, low birth weight, fetal distress, and greater likelihood of neonatal intensive care admission [1]. The complex interplay between immune-mediated placental dysfunction, systemic maternal physiology, and the potential for limited direct viral effects underscores the need for an integrated mechanistic understanding of COVID-19 in pregnancy [8]. To contextualise current knowledge and identify areas requiring further investigation, this review synthesises molecular, immunological, pathological and clinical evidence, drawing on published experimental, translational, and computational analyses to elucidate the multifaceted interaction between SARS-CoV-2 and pregnancy-specific biology.

## 2. Molecular Basis of SARS-CoV-2 Entry and Host Receptor Expression in Pregnancy

### 2.1. SARS-CoV-2 Viral Architecture and Entry Mechanisms

SARS-CoV-2 is an enveloped, positive-sense RNA virus of the Betacoronavirus lineage. Its ~30 kb genome encodes the structural spike (S), envelope (E), membrane (M), and nucleocapsid (N) proteins, along with 16 non-structural proteins critical for replication and immune evasion. The spike glycoprotein mediates host cell entry and is composed of an S1 subunit containing the receptor-binding domain (RBD) and an S2 subunit responsible for membrane fusion [10,11].

Spike attachment to ACE2 is facilitated by residues N501, Y505, Q493, Y489, and K417, forming a high-affinity binding interface. Following attachment, host proteases cleave the spike at the S1/S2 boundary. Importantly, SARS-CoV-2 possesses a unique furin cleavage site (PRRAR) not present in SARS-CoV, which enhances S1/S2 priming and increases transmissibility [12,13,14].

Variant-specific mutations influence viral affinity for ACE2. For example, N501Y (Alpha, Beta, Gamma), L452R/T478K (Delta), and Q498R combined with N501Y (Omicron) significantly increase RBD–ACE2 binding affinity. SARS-CoV-2 also employs multiple immune-evasion strategies. NSP1 suppresses host translation, ORF6 blocks STAT1 nuclear import, and ORF3a activates the NLRP3 inflammasome, enabling viral persistence [15,16,17].

### 2.2. Expression of Viral Entry Receptors in Placental and Maternal Tissues

#### 2.2.1. ACE2 Expression

ACE2 is expressed in syncytiotrophoblasts, cytotrophoblasts, extravillous trophoblasts, decidual stromal cells, and fetal endothelial cells. Its expression is highest in early pregnancy and declines toward term [18].

#### 2.2.2. TMPRSS2 Expression

TMPRSS2 (transmembrane serine protease 2), which is required for cell-surface SARS-CoV-2 Spike priming, exhibits minimal expression across most placental cell types, particularly in early gestation. This restricted distribution significantly limits canonical Spike activation at the maternal–fetal interface and is considered a major reason for the reduced susceptibility of placental tissues to productive viral entry [19]. In addition to ACE2, placental tissues express several auxiliary entry mediators, including neuropilin-1 (NRP1), cathepsin L (CTSL), furin (FURIN), and basigin (BSG, also known as CD147), each of which may contribute to alternative infection pathways or influence viral internalisation [20]. NRP1 has been shown to enhance SARS-CoV-2 uptake, CTSL supports endosomal entry pathways, FURIN is abundantly expressed in syncytiotrophoblasts and facilitates Spike activation through S1/S2 cleavage, and BSG has been proposed as a potential co-receptor in facilitating viral adherence [19,20].

#### 2.2.3. Spatial Localization

Spatial localisation studies using immunohistochemistry demonstrate distinct distribution patterns of these entry factors within placental structures. ACE2 and NRP1 are predominantly localised to the apical membrane of syncytiotrophoblasts, aligning them directly at the maternal–fetal interface. CTSL is primarily situated within cytotrophoblast endosomal compartments, consistent with its role in endosomal fusion pathways, while FURIN is expressed both in syncytiotrophoblasts and villous stromal cells, supporting its involvement in intracellular and extracellular proteolytic activation [21].

Despite the presence of these receptors and cofactors, widespread placental infection remains uncommon. Several mechanisms contribute to this limited viral permissiveness, including low co-expression of ACE2 and TMPRSS2 within the same placental cells, polarised receptor distribution that restricts viral access, strong trophoblast-intrinsic interferon (IFN) responses that generate an antiviral environment, and limited availability of endosomal machinery required for efficient coronavirus replication. These factors collectively suggest that susceptibility during pregnancy arises less from high placental viral burden and more from systemic inflammation and endothelial injury associated with maternal SARS-CoV-2 infection [21,22].

## 3. Immunological and Inflammatory Responses in Pregnancy During SARS-CoV-2 Infection

### 3.1. Gestational Immune Modulation and Viral Susceptibility

Pregnancy is characterized by dynamic immunological transitions that support fetal tolerance while maintaining pathogen defense. The first trimester is dominated by pro-inflammatory Th1-type responses necessary for implantation and placentation, while the second trimester shifts toward a tolerogenic state, with increased regulatory T cells (Tregs), IL-10, and Th2 cytokines. The third trimester again becomes pro-inflammatory to support labor initiation. This cyclical modulation creates windows of vulnerability where antiviral immunity can become attenuated, particularly during mid-gestation when interferon responses are suppressed [23,24].

### 3.2. Maternal Immune Response to SARS-CoV-2

Upon SARS-CoV-2 infection, pregnant individuals exhibit exaggerated inflammatory signaling compared to non-pregnant adults. Studies show elevated levels Interleukin-6 (IL-6), Tumor necrosis factor-alpha (TNF-α), C-X-C motif chemokine ligand 8/Interleukin-8 (CXCL8/IL-8), Interleukin-1 beta (IL-1β), Interferon-gamma (IFN-γ) [25,26]. These cytokines correlate with severe maternal outcomes. Placental immune cells, including macrophages (Hofbauer cells) and decidual NK cells, show activation signatures with increased interferon-stimulated genes (ISGs) such as MX1, OAS1, IFITM3, contributing to a heightened antiviral but often dysregulated response [27].

### 3.3. Interferon Pathway Activation and Dysregulation

Although SARS-CoV-2 typically suppresses type I interferon (IFN-I) responses through viral proteins (NSP1, ORF6), placental tissues mount a stronger IFN response than other organs. Trophoblasts constitutively express IFN-λ and have intrinsic antiviral mechanisms [28].

However, systemic maternal IFN activation can impair trophoblast differentiation, angiogenesis and spiral artery remodeling leading to placental malperfusion and adverse pregnancy outcomes [29].

### 3.4. Endothelial Dysfunction and Thrombo-Inflammation in Pregnancy

SARS-CoV-2 infection contributes to widespread endothelial injury through multiple mechanisms, including complement activation, the release of neutrophil extracellular traps (NETs), formation of microthrombi, and diffuse vascular inflammation [30]. Pregnancy is inherently a hypercoagulable state characterised by increased coagulation factor activity, reduced fibrinolysis, and enhanced platelet reactivity. When this physiological state is combined with SARS-CoV-2–mediated endothelial disruption, the risk of coagulopathy is amplified, leading to elevated D-dimer concentrations, microvascular thrombosis, and an increased likelihood of developing preeclampsia-like clinical presentations [31]. Corresponding placental pathology frequently demonstrates features of maternal vascular malperfusion, extensive fibrinoid deposition, intervillous thrombi, and decidual arteriopathy [32,33]. These abnormalities are observed even in the absence of detectable viral RNA within placental tissues, indicating that systemic inflammation and immune-mediated endothelial dysfunction, rather than direct viral infection, are the primary drivers of placental injury in COVID-19-affected pregnancies.

### 3.5. Cytokine Storm Amplification in Pregnancy

Severe SARS-CoV-2 infection in pregnancy is characterised by pronounced cytokine release, particularly involving interleukin-6 (IL-6) and tumour necrosis factor-alpha (TNF-α), which act as major mediators of systemic inflammation [34]. Elevated concentrations of these cytokines disrupt syncytiotrophoblast barrier integrity, impair placental progesterone synthesis, and compromise uteroplacental perfusion. These alterations collectively reduce placental blood flow and heighten the risk of fetal distress, even in the absence of substantial viral load. The resulting inflammatory amplification is strongly associated with increased rates of preterm birth, fetal growth restriction, hypertensive disorders of pregnancy, and emergency caesarean delivery [35]. These findings underscore that cytokine-driven immune dysregulation, rather than direct viral replication, is a primary determinant of adverse obstetric outcomes in severe COVID-19.

### 3.6. Integration of Immune Dynamics with SARS-CoV-2 Pathogenesis

**Figure 1:**
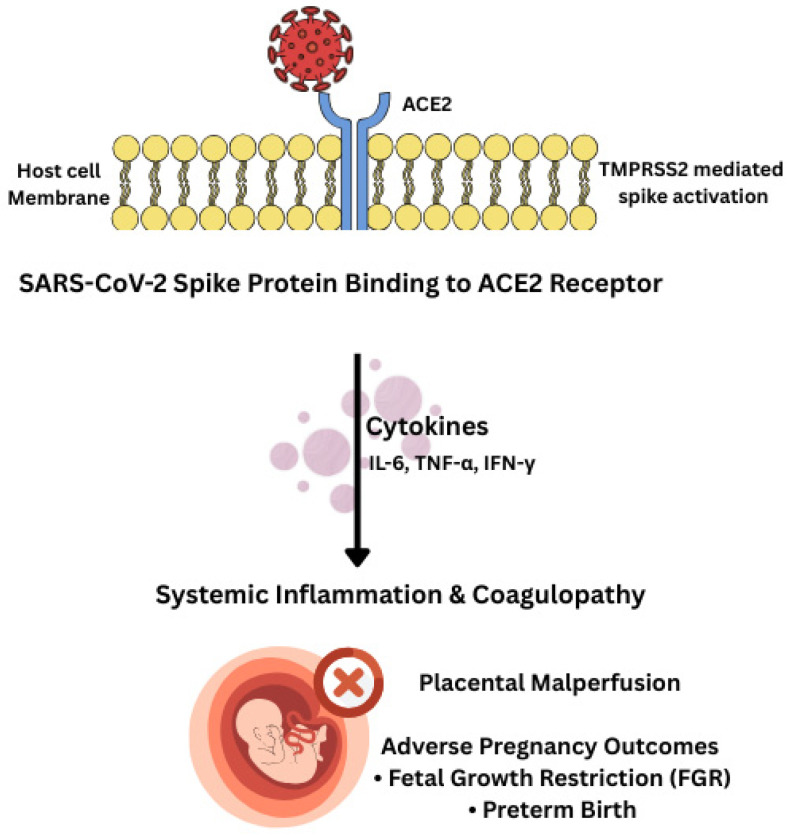
Gestational Modulation of SARS-CoV-2 Entry and Immune Activation at the Maternal–Fetal Interface.

The combination of pregnancy-specific immune tolerance, gestational shifts in macrophage/NK activity, reduced antiviral signaling, and heightened inflammatory susceptibility creates a unique immunological profile that amplifies SARS-CoV-2 pathogenesis. The placenta, though rarely infected, becomes collateral damage due to systemic inflammatory cascades and endothelial dysfunction. This immunological landscape forms the mechanistic basis for the maternal and fetal outcomes described in subsequent sections (Figure 1) [36].

The schematic illustrates the sequential progression from viral spike–ACE2 binding and protease-mediated priming to systemic immune activation, characterized by elevated cytokines. These events drive endothelial dysfunction and coagulopathy, culminating in placental malperfusion and adverse clinical outcomes, including preterm birth, fetal growth restriction (FGR), and preeclampsia-like syndrome. Note: Color variations are utilized for visual clarity and do not indicate functional categories.

## 4. Placental and Maternal Pathophysiology in SARS-CoV-2 Infection

### 4.1. Placental Structural and Functional Alterations

Although confirmed, diffuse placental infection with SARS-CoV-2 is uncommon, multiple studies consistently describe characteristic histopathological abnormalities in placentas from infected pregnancies. These include maternal vascular malperfusion (MVM), intervillous thrombosis, decidual arteriopathy, retroplacental haemorrhage, increased fibrinoid necrosis, and features such as chorangiosis and villous hypoplasia [37]. These lesions reflect disturbances in perfusion and vascular integrity and are strongly associated with maternal systemic inflammation, endothelial injury and a hypercoagulable physiological state, rather than with direct or widespread viral replication within placental tissue. The pattern of pathology therefore aligns with the concept that maternal immune activation, rather than placental viral burden, is the predominant driver of these structural alterations [38].

### 4.2. Trophoblast Injury and Barrier Disruption

The syncytiotrophoblast, which constitutes the primary barrier and exchange surface at the maternal–fetal interface, demonstrates several ultrastructural and functional changes in pregnancies affected by SARS-CoV-2. Reports describe mitochondrial swelling, increased apoptotic activity, microvillous damage and reduced expression of key nutrient transporters [39], even in cases where viral RNA is not detectable. These alterations impair multiple aspects of placental function, including amino acid transport, fatty acid transfer, steroidogenic capacity and angiogenic signalling pathways [40]. The cumulative effect of these disruptions contributes to fetal growth impairment, metabolic disturbances and broader materno-fetal complications associated with COVID-19 during pregnancy.

### 4.3. Angiogenic Imbalance and Preeclampsia-like Manifestations

SARS-CoV-2 infection is strongly associated with dysregulation of angiogenic pathways, characterised by increased circulating levels of soluble fms-like tyrosine kinase-1 (sFlt-1; FLT1) and soluble endoglin (sEng), reduced concentrations of placental growth factor (PlGF), and impaired vascular endothelial growth factor (VEGF) signalling [41]. This anti-angiogenic shift closely mirrors the molecular profile observed in preeclampsia and reflects significant endothelial disturbance. Inflammatory and cytokine-driven endothelial dysfunction further contributes to the development of hypertension, proteinuria, hepatocellular injury and elevated uterine artery resistance [42]. These physiological alterations correspond with the placental histopathological findings described in COVID-19 pregnancies and offer a mechanistic explanation for the increased incidence of preeclampsia-like syndromes observed in infected pregnant individuals.

### 4.4. Complement Activation and Coagulopathy

SARS-CoV-2 triggers widespread activation of the alternative complement pathway, lectin pathway, and the terminal complement cascade, including formation of the membrane attack complex (C5b-9) [43]. Activation of these pathways contributes to endothelial damage, microvascular thrombosis and local inflammatory amplification. As pregnancy itself is a hypercoagulable state, SARS-CoV-2 superimposes an additional thrombotic burden, significantly increasing the risk of placental microthrombi, intervillous thrombosis, pulmonary embolism and, in rare cases, disseminated intravascular coagulation [44]. These coagulopathic events impair uteroplacental perfusion and compromise fetal oxygen delivery, thereby contributing to adverse pregnancy outcomes.

### 4.5. Steroidogenesis and Endocrine Disruption

The placenta functions as a major endocrine organ, producing progesterone via 3β-hydroxysteroid dehydrogenase type 1 (HSD3B1), estradiol via CYP19A1 (aromatase), placental lactogen (CSH1) and various placental growth hormone isoforms. Inflammatory cytokines such as IL-6 (interleukin-6) and TNF-α (tumour necrosis factor-alpha) downregulate the expression of these steroidogenic enzymes, resulting in reduced progesterone synthesis, impaired decidualisation, increased uterine contractility and a heightened risk of preterm labour. Importantly, even in the absence of direct viral invasion, cytokine-driven endocrine disruption can lead to clinically significant obstetric complications, emphasising the indirect pathways through which SARS-CoV-2 affects pregnancy [45]. Beyond steroidogenesis, progesterone and estrogen exert critical immunomodulatory functions. Progesterone promotes regulatory T-cell differentiation, suppresses NF-κB–mediated inflammatory signaling, and attenuates IL-6 production. Estrogen modulates ACE2 expression and interferon responses, potentially influencing SARS-CoV-2 susceptibility and immune activation dynamics during pregnancy.

### 4.6. Impact on Fetal Growth and Development

The combined effects of reduced uteroplacental perfusion, angiogenic imbalance, trophoblast injury and endocrine disturbances contribute to increased rates of fetal growth restriction (FGR), preterm birth, low birth weight and fetal distress requiring urgent delivery. These outcomes have been documented even in pregnancies with mild maternal disease, indicating that subtle placental alterations can exert measurable effects on fetal development [46]. The constellation of growth and developmental impairments underscores the vulnerability of the fetoplacental unit to inflammatory and vascular perturbations during SARS-CoV-2 infection.

### 4.7. Mechanistic Integration

**Figure 2:**
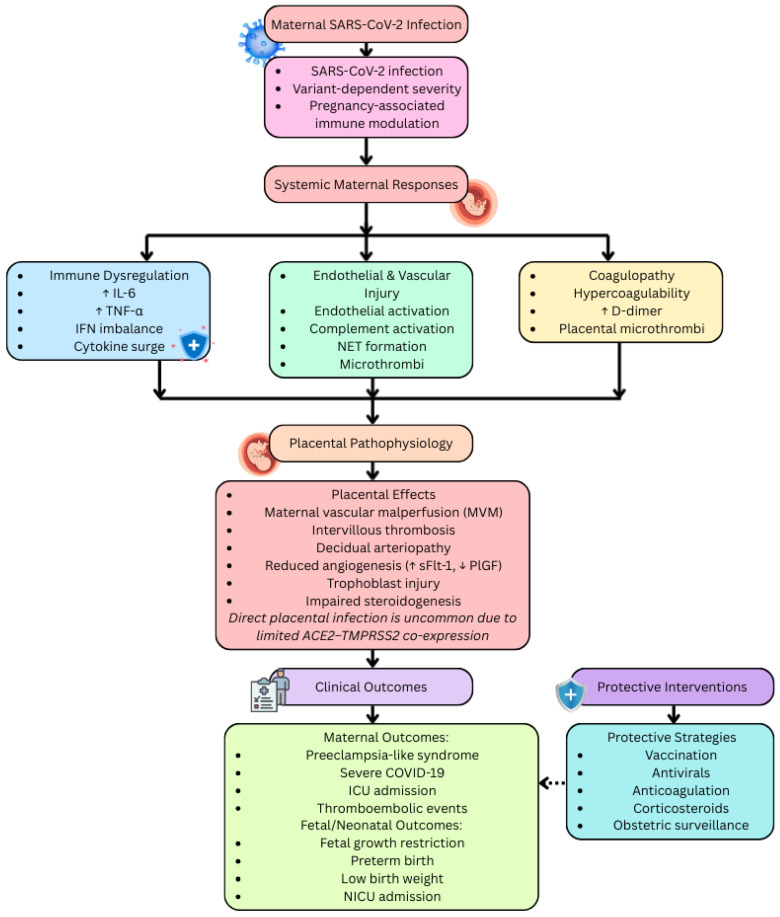
Mechanistic overview of SARS-CoV-2-associated maternal and placental pathophysiology during pregnancy. Note: Different colors are utilized for visual clarity and have no specific functional significance.

Overall, the dominant mechanism underlying placental and maternal pathology in SARS-CoV-2 infection is immunologically mediated vascular injury rather than widespread viral tropism. The pathological sequence involves systemic cytokine elevation following maternal infection, leading to endothelial dysfunction and complement activation, which subsequently produces placental vascular malperfusion and infarction. These vascular insults cause trophoblast stress, impair nutrient and hormone exchange, and generate angiogenic imbalance that culminates in preeclampsia-like features. The resulting fetal stress contributes to preterm birth and fetal growth restriction [37,38,39,40,41,42,43,44,45,46]. This integrative mechanistic framework demonstrates how inflammation, coagulation abnormalities, and endothelial injury collectively drive the adverse outcomes observed in COVID-19–affected pregnancies (Figure 2).

The schematic depicts the progression from viral infection and systemic maternal responses—characterized by immune dysregulation, endothelial injury, and coagulopathy—to placental pathophysiology, including maternal vascular malperfusion (MVM) and trophoblast injury. Clinical outcomes and protective strategies are highlighted.

Maternal SARS-CoV-2 infection induces systemic immune dysregulation, endothelial injury, and thrombo-inflammatory signaling, which converge on the placenta to cause vascular malperfusion, angiogenic imbalance, trophoblast dysfunction, and endocrine disruption. These indirect mechanisms, rather than direct placental infection, underlie adverse maternal and fetal outcomes including preeclampsia-like syndromes, fetal growth restriction, and preterm birth. Preventive and therapeutic interventions such as vaccination, antiviral therapy, anticoagulation, and obstetric surveillance mitigate these pathological cascades and improve pregnancy outcomes. Abbreviations used include ACE2: angiotensin-converting enzyme 2; TMPRSS2: transmembrane serine protease 2; IL-6: interleukin-6; TNF-α: tumor necrosis factor-alpha; IFN: interferon; NETs: neutrophil extracellular traps; MVM: maternal vascular malperfusion; sFlt-1: soluble fms-like tyrosine kinase-1; PlGF: placental growth factor; ICU: intensive care unit; NICU: neonatal intensive care unit.

## 5. Clinical Outcomes in Pregnancies Complicated by SARS-CoV-2 Infection

### 5.1. Maternal Clinical Outcomes

Pregnant individuals infected with SARS-CoV-2 exhibit a significantly higher risk of severe clinical disease compared with non-pregnant women of reproductive age. Large meta-analyses and multinational cohort studies consistently demonstrate increased rates of severe COVID-19, intensive care unit (ICU) admission, invasive mechanical ventilation, extracorporeal membrane oxygenation (ECMO) support, and maternal mortality among pregnant patients [26]. This heightened vulnerability is largely attributed to pregnancy-associated physiological adaptations, including reduced functional residual capacity, increased oxygen consumption, elevated diaphragmatic positioning, and pregnancy-specific alterations in interferon-mediated antiviral signaling pathways [26]. These factors collectively reduce pulmonary reserve and impair effective viral clearance, thereby predisposing pregnant individuals to respiratory decompensation during SARS-CoV-2 infection. The presence of comorbid conditions such as obesity, gestational diabetes mellitus, and hypertensive disorders of pregnancy further amplifies disease severity and adverse maternal outcomes [47].

### 5.2. Obstetric Complications

SARS-CoV-2 infection during pregnancy is associated with an increased incidence of obstetric complications, particularly among symptomatic individuals and those requiring hospitalization. Large population-based studies and systematic reviews report significantly elevated risks of pre-eclampsia or pre-eclampsia-like syndromes, spontaneous and iatrogenic preterm labor, premature rupture of membranes, cesarean delivery, and postpartum hemorrhage in cases of severe maternal infection [48]. Preterm birth in the context of COVID-19 is frequently medically indicated, driven by worsening maternal hypoxia, progressive hypertensive disease, or non-reassuring fetal status rather than spontaneous preterm labor alone [48]. These findings underscore the multifactorial nature of obstetric risk in SARS-CoV-2–affected pregnancies, where maternal systemic inflammation, endothelial dysfunction, and placental malperfusion converge to influence clinical decision-making and perinatal outcomes.

### 5.3. Fetal and Neonatal Outcomes

Although vertical transmission of SARS-CoV-2 is uncommon, adverse fetal outcomes are frequently reported and are primarily attributed to placental dysfunction, systemic maternal inflammation, and impaired uteroplacental perfusion rather than direct fetal infection. Observational studies and population-based analyses have demonstrated an increased incidence of fetal growth restriction (FGR), low birth weight (LBW), higher rates of neonatal intensive care unit (NICU) admission, and a modestly elevated risk of stillbirth, particularly in cases of severe maternal disease [49]. These outcomes correlate strongly with maternal systemic inflammatory burden—characterised by elevated interleukin-6 (IL-6) and tumour necrosis factor-alpha (TNF-α)—as well as placental vascular malperfusion, complement activation, and infection occurring during late gestation, rather than maternal viral load alone [50]. Collectively, these findings support a placental-mediated mechanism of fetal compromise in SARS-CoV-2-affected pregnancies.

### 5.4. Neonatal Infection and Vertical Transmission

Confirmed vertical transmission of SARS-CoV-2 is rare, with reported rates below 2% across large cohort studies. The infrequency of transplacental infection is attributed to limited co-expression of angiotensin-converting enzyme 2 (ACE2) and transmembrane serine protease 2 (TMPRSS2) within placental tissues, robust trophoblast-mediated antiviral defenses including type III interferon (IFN-λ) signaling, and the expression of interferon-induced transmembrane (IFITM) proteins that restrict viral fusion and replication [51]. As a result, most neonates born to infected mothers test negative for viral RNA at birth and, when infection occurs postnatally, typically exhibit mild or asymptomatic disease courses [51].

### 5.5. Timing of Infection and Outcome Severity

Gestational timing of maternal SARS-CoV-2 infection significantly modulates clinical outcomes. First-trimester infection has not been conclusively associated with increased miscarriage risk, although subtle effects on early placental development cannot be excluded based on current evidence. Second-trimester infection appears to confer the greatest susceptibility to inflammatory placental injury, with higher rates of fetal growth restriction and placental vascular abnormalities reported. In contrast, third-trimester infection is associated with the highest risk of maternal respiratory deterioration, medically indicated preterm birth, preeclampsia-like manifestations, and increased NICU admissions due primarily to prematurity [52,53]. These temporal differences underscore the dynamic vulnerability of the maternal–fetal unit across gestation.

### 5.6. Integration of Clinical Outcomes with Pathophysiology

The clinical manifestations observed in SARS-CoV-2–affected pregnancies arise from a convergence of interconnected pathophysiological mechanisms. Maternal immune dysregulation leads to excessive cytokine signaling and endothelial injury, which in turn precipitate placental vascular malperfusion and impaired oxygen and nutrient transfer. Concurrent endocrine dysfunction, including reduced progesterone synthesis, contributes to altered uterine contractility, while angiogenic imbalance promotes hypertensive disorders of pregnancy. Thrombo-inflammatory processes further exacerbate microvascular pathology, and physiological respiratory limitations inherent to pregnancy increase the likelihood of hospitalization and intensive care requirements [54]. Maternal disease severity remains the strongest predictor of adverse fetal and neonatal outcomes, highlighting the importance of early detection, close monitoring, and preventive strategies such as vaccination.

## 6. Systems-Level Insights into SARS-CoV-2-Induced Placental Dysfunction

Advances in computational biology and systems-level analyses have substantially enhanced understanding of SARS-CoV-2-associated placental pathology by integrating transcriptomic, proteomic, and protein–protein interaction (PPI) datasets derived from infected maternal and placental tissues. Multiple published in silico studies have demonstrated that SARS-CoV-2–responsive gene networks in pregnancy converge on immune regulation, vascular integrity, angiogenesis, and endocrine signaling pathways, providing mechanistic insight into the indirect effects of maternal infection on placental function even in the absence of direct viral replication [55,56,57,58,59].

Network-based analyses using platforms such as STRING, Cytoscape, and GeneMANIA consistently identify angiotensin-converting enzyme 2 (ACE2), interleukin-6 (IL6), tumor necrosis factor (TNF), vascular endothelial growth factor A (VEGFA), Fms-like tyrosine kinase 1 (FLT1), and nuclear factor kappa B (NF-κB) as central hubs linking antiviral responses with inflammatory, thrombotic, and angiogenic pathways. These hub nodes exhibit extensive functional connectivity, suggesting that SARS-CoV-2 infection amplifies pre-existing pregnancy-associated signaling programs rather than introducing novel placental tropism [60,61,62].

Transcriptomic profiling of placental tissues from SARS-CoV-2–exposed pregnancies reveals upregulation of interferon-stimulated genes (ISGs), chemokines (CCL2, CXCL10), and complement-related transcripts, alongside suppression of genes involved in trophoblast differentiation, lipid transport, and steroidogenesis. In silico pathway enrichment analyses consistently implicate dysregulation of PI3K–Akt signaling, MAPK cascades, hypoxia-inducible factor-1 (HIF-1) signaling, and transforming growth factor-beta (TGF-β) pathways, all of which are essential for placental vascular development and maternal–fetal exchange [62,63].

Computational modeling of endothelial–immune interactions further supports a central role for thrombo-inflammatory signaling in SARS-CoV-2-associated pregnancy complications. Published network simulations demonstrate functional overlap between COVID-19-induced cytokine signatures and molecular profiles characteristic of preeclampsia, including elevated soluble fms-like tyrosine kinase-1 (sFlt-1), endoglin (ENG), and reduced placental growth factor (PlGF) signaling. These findings align with clinical observations of preeclampsia-like syndromes in infected pregnancies and reinforce the concept that SARS-CoV-2 acts as a systemic vascular stressor rather than a placental cytopathic agent [64,65].

Several integrative in silico studies have also examined the structural and functional consequences of spike protein interactions with ACE2 in reproductive tissues, confirming high-affinity binding while simultaneously highlighting limited co-expression of essential proteases such as TMPRSS2 in placental cell populations. Computational predictions therefore support a model in which placental susceptibility is determined not by viral entry efficiency alone but by downstream immune and vascular signaling cascades activated in response to maternal infection [58,66].

Importantly, systems biology approaches integrating maternal blood transcriptomes with placental gene expression profiles reveal strong correlations between maternal inflammatory load and placental vascular injury signatures, independent of detectable placental viral RNA [67]. These data reinforce the hypothesis that maternal immune dysregulation serves as the primary driver of placental pathology, fetal growth restriction, and adverse obstetric outcomes in SARS-CoV-2–affected pregnancies.

Collectively, published in silico and translational studies provide compelling evidence that SARS-CoV-2-associated placental dysfunction arises from convergence of immune activation, endothelial injury, angiogenic imbalance, and endocrine disruption. By contextualizing molecular alterations within pregnancy-specific physiological frameworks, these systems-level analyses offer critical insights into disease mechanisms and identify potential biomarkers and therapeutic targets for risk stratification and clinical management.

## 7. Vaccination, Therapeutics, and Current Management Guidelines in Pregnant Individuals with SARS-CoV-2 Infection

### 7.1. Safety and Effectiveness of COVID-19 Vaccination in Pregnancy

Multiple large-scale population-based studies and meta-analyses have established that mRNA COVID-19 vaccines, including BNT162b2 (Pfizer–BioNTech) and mRNA-1273 (Moderna), are safe, highly effective, and strongly recommended during pregnancy [68]. Vaccination during gestation is not associated with an increased risk of miscarriage, congenital anomalies, preterm birth, placental abruption, or stillbirth. In contrast, vaccinated pregnant individuals demonstrate a significant reduction in SARS-CoV-2 infection rates, hospitalization, intensive care unit (ICU) admission, and progression to severe disease. Importantly, vaccine effectiveness has been maintained across circulating variants, including Delta and Omicron, and vaccination partially corrects pregnancy-associated immunological vulnerabilities by enhancing neutralizing antibody responses and restoring attenuated interferon-mediated antiviral immunity [69].

### 7.2. Booster Dose Benefits

Administration of booster doses during pregnancy has been shown to substantially augment humoral immunity and cross-variant protection, particularly against Omicron subvariants. Boosted pregnant individuals exhibit three- to four-fold higher neutralizing antibody titers compared with those receiving only a two-dose primary series [70]. Booster vaccination is associated with further reductions in maternal hospitalization, ICU admission, preterm birth, and severe neonatal disease, reinforcing its role as a critical preventive intervention during ongoing viral evolution.

### 7.3. Transplacental Transfer of Antibodies and Neonatal Immunity

Maternal COVID-19 vaccination results in efficient transplacental transfer of immunoglobulin G (IgG), conferring passive immunity to the neonate during early life. Cord blood neutralizing antibody titers closely mirror maternal levels, indicating effective placental transport [71]. Antibody transfer efficiency is highest when vaccination occurs during the second trimester or early third trimester, allowing sufficient time for IgG1 maturation and placental transfer. Infants born to vaccinated mothers demonstrate significantly lower rates of COVID-19-related hospitalization, highlighting the dual maternal–neonatal benefit of antenatal immunization.

### 7.4. Pharmacologic Management of COVID-19 in Pregnancy

#### 7.4.1. Antiviral Therapy

Antiviral agents are recommended for symptomatic pregnant individuals at increased risk of disease progression. Nirmatrelvir–ritonavir (Paxlovid) is currently recommended for eligible pregnant patients, with no teratogenic signals observed from ritonavir-containing regimens. Remdesivir has also demonstrated a favorable safety profile in pregnancy, supported by registry data, and reduces progression to severe disease when administered early in the clinical course [72].

#### 7.4.2. Corticosteroids

Systemic corticosteroids are indicated for pregnant individuals with moderate to severe COVID-19 requiring supplemental oxygen. Dexamethasone has been shown to reduce mortality in severe disease, as demonstrated in the RECOVERY trial. While dexamethasone is effective for maternal treatment, betamethasone is preferred when fetal lung maturation is also indicated [73].

#### 7.4.3. Anticoagulation

Given the intrinsically hypercoagulable state of pregnancy compounded by SARS-CoV-2-associated coagulopathy, prophylactic low-molecular-weight heparin (LMWH) is recommended for hospitalized pregnant patients with COVID-19. LMWH administration reduces the risk of venous thromboembolism, placental microthrombi formation, and maternal morbidity, thereby improving maternal and placental vascular outcomes [74].

#### 7.4.4. Monoclonal Antibodies

Monoclonal antibody therapies, when active against circulating variants, have been shown to reduce viral load and hospitalization risk in pregnant individuals. Agents such as sotrovimab and casirivimab–imdevimab have historically been used, with available safety data demonstrating no increase in adverse pregnancy outcomes. However, clinical utility varies with emerging variants, and recommendations continue to evolve accordingly [75].

### 7.5. Obstetric Management and Clinical Guidelines

Guidelines from major international organizations, including the American College of Obstetricians and Gynecologists (ACOG, Washington, DC, USA), Royal College of Obstetricians and Gynaecologists (RCOG, London, UK), World Health Organization (WHO), and Centers for Disease Control and Prevention (CDC, Atlanta, GA, USA), emphasize individualized, multidisciplinary care for pregnant individuals with SARS-CoV-2 infection. Recommended practices include continuous monitoring of maternal oxygen saturation, avoidance of unnecessary early delivery, enhanced fetal surveillance when maternal condition deteriorates, and early evaluation for preeclampsia in third-trimester infections. Vaginal delivery is permitted unless maternal respiratory compromise necessitates operative intervention. Breastfeeding is encouraged with appropriate infection-control precautions, including masking and hand hygiene. Across all guidelines, vaccination and timely antiviral therapy remain the most effective protective strategies [76,77,78].

### 7.6. Synthesis of Therapeutic Evidence

Collectively, current therapeutic and preventive strategies align with mechanistic insights derived from clinical, molecular, and systems-level studies [64]. Vaccination mitigates systemic hyperinflammation, thereby reducing placental malperfusion, preeclampsia-like syndromes, and preterm birth. Antiviral therapies limit viral replication and attenuate interferon-driven immunopathology, while anticoagulation addresses coagulopathic and angiogenic disturbances observed in COVID-19-affected pregnancies. Efficient transplacental transfer of vaccine-induced IgG further substantiates the low fetal risk and substantial neonatal benefit associated with maternal immunization. Together, a combined strategy encompassing vaccination, early pharmacologic intervention, and vigilant obstetric surveillance offers optimal outcomes for both mother and fetus.

## 8. Discussion

Pregnancy represents a uniquely vulnerable physiological state in which immunological modulation, endocrine adaptations, and cardiovascular remodeling intersect to support fetal development while simultaneously altering host responses to infection. This review synthesizes current clinical, molecular, and translational evidence to elucidate how SARS-CoV-2 infection disrupts this finely balanced system. Accumulating data consistently demonstrate that direct placental infection by SARS-CoV-2 is uncommon, likely due to limited co-expression of angiotensin-converting enzyme 2 (ACE2) and transmembrane serine protease 2 (TMPRSS2), polarized receptor distribution, and robust trophoblast-intrinsic antiviral defenses. Nevertheless, adverse obstetric and perinatal outcomes remain disproportionately elevated, indicating that indirect mechanisms predominate in COVID-19-associated pregnancy complications [79].

In examining placental vulnerability to Severe acute respiratory syndrome coronavirus 2, this review adopts an integrated framework that begins with viral entry biology and extends through immune activation to placental dysfunction and clinical outcomes. Although confirmed placental infection remains relatively infrequent, the coordinated effects of Spike–ACE2 interaction, co-factor availability, and gestational receptor distribution establish a theoretical window for viral engagement at the maternal–fetal interface. More importantly, SARS-CoV-2–mediated immune activation—characterised by interferon dysregulation, cytokine amplification, endothelial injury, and complement activation—appears to represent the principal driver of placental vulnerability. Within this context, placental physiological adaptations during pregnancy do not merely serve as background features but actively shape the magnitude and consequences of immune-mediated injury. Thus, viral entry pathways and downstream immune responses function as the initiating events, while vascular malperfusion, endocrine perturbation, and adverse obstetric outcomes represent the integrated biological sequelae.

### 8.1. Maternal Immune Dysregulation as the Central Driver of Pathology

Evidence across clinical cohorts, placental histopathology, and transcriptomic analyses supports maternal systemic inflammation as the principal mediator of SARS-CoV-2–related placental dysfunction. Elevated circulating cytokines, particularly interleukin-6 (IL-6) and tumor necrosis factor-alpha (TNF-α), promote endothelial activation, complement cascade engagement, and thrombo-inflammatory signaling, resulting in widespread vascular injury. These inflammatory processes extend to the maternal–fetal interface, where they impair trophoblast function, disrupt placental perfusion, and precipitate maternal vascular malperfusion, even in the absence of detectable placental viral RNA. This inflammatory paradigm explains the frequent observation of preeclampsia-like syndromes, fetal growth restriction, and medically indicated preterm birth in affected pregnancies.

### 8.2. Angiogenic and Endothelial Imbalance Links Molecular Changes to Clinical Phenotypes

A defining feature of SARS-CoV-2-affected pregnancies is the disruption of angiogenic homeostasis. Elevated soluble fms-like tyrosine kinase-1 (sFlt-1) and endoglin (sEng), coupled with reduced placental growth factor (PlGF), mirror the molecular profile of preeclampsia and correlate with Doppler abnormalities, placental infarction, and hypertensive disorders. These angiogenic perturbations are exacerbated by endothelial injury and complement activation, collectively impairing uteroplacental blood flow and fetal oxygen delivery. Importantly, these changes appear to reflect immune-mediated vascular stress rather than direct viral cytopathicity, reinforcing the concept that SARS-CoV-2 acts primarily as a systemic vascular and inflammatory insult during pregnancy [80].

### 8.3. Endocrine Disruption and Trophoblast Dysfunction

Placental endocrine function is highly sensitive to inflammatory signaling. Pro-inflammatory cytokines suppress key steroidogenic enzymes, including aromatase (CYP19A1) and 3β-hydroxysteroid dehydrogenase (HSD3B1), resulting in reduced progesterone and estrogen synthesis. This endocrine disruption compromises decidualization, increases uterine contractility, and heightens susceptibility to preterm labor. Concurrent trophoblast injury, characterized by mitochondrial dysfunction, apoptosis, and reduced nutrient transporter expression, further limits fetal growth and metabolic stability. Together, immune-mediated endocrine and trophoblast dysfunction provide a mechanistic basis for adverse fetal outcomes observed even in mild or moderate maternal disease [81].

### 8.4. Emerging Variants, Immune Evasion, and Potential Implications for Placental Tropism

Successive SARS-CoV-2 variants have acquired mutations in the Spike protein and non-structural regions that enhance transmissibility, ACE2 binding affinity, and immune evasion. Variants such as Delta and Omicron demonstrate increased ACE2 affinity through receptor-binding domain mutations, theoretically improving entry into ACE2-expressing placental trophoblasts.

However, placental susceptibility depends not only on ACE2 binding but also on TMPRSS2 co-expression, receptor polarisation, and intrinsic trophoblast antiviral defences. Robust interferon responses and limited cofactor availability likely continue to restrict productive placental infection, even with higher-affinity variants.

Mutations that enhance interferon suppression and immune escape may instead amplify systemic maternal inflammation, endothelial activation, and complement dysregulation—mechanisms linked to placental vascular injury and adverse pregnancy outcomes. Current evidence does not indicate variant-specific increases in confirmed placental infection; rather, maternal inflammatory severity remains the dominant determinant of placental dysfunction. Continued surveillance is warranted as viral evolution progresses.

### 8.5. Clinical Implications and Preventive Strategies

The convergence of immune activation, vascular injury, angiogenic imbalance, and endocrine disruption underscores the importance of preventive and early therapeutic interventions. Vaccination has emerged as the most effective strategy for mitigating maternal morbidity, reducing systemic inflammation, and preventing severe disease [82]. By attenuating the inflammatory cascade, vaccination indirectly preserves endothelial function, placental perfusion, and angiogenic balance, thereby reducing the incidence of preeclampsia-like syndromes and preterm birth. Antiviral therapies, corticosteroids, and anticoagulation further complement this approach when applied judiciously, emphasizing the need for individualized, guideline-based clinical management. Importantly, placental vulnerability cannot be interpreted independently of maternal clinical trajectory. Emerging data suggest that the severity and timing of maternal SARS-CoV-2 infection significantly influence fetal and obstetric outcomes. Severe maternal disease, high systemic inflammatory burden, and pre-existing comorbidities such as obesity, hypertension, or metabolic dysfunction appear to amplify risks of placental vascular malperfusion and preterm birth. Additionally, infection during early gestation may carry different immunological and developmental implications compared to third-trimester exposure, when placental maturation and immune tolerance mechanisms differ substantially. These observations highlight that placental injury reflects not only viral biology but also host clinical context and disease dynamics.

### 8.6. Knowledge Gaps

Despite rapid advances, several critical gaps remain in understanding SARS-CoV-2 infection during pregnancy. First, the placental and vascular effects of emerging variants, including XBB lineages and recently circulating strains, remain incompletely characterized. Second, high-resolution single-cell and spatial transcriptomic studies across gestation are limited, restricting insight into cell-type-specific susceptibility and microregional placental injury. Third, the molecular distinction between classical preeclampsia and COVID-19–associated hypertensive disorders remains unclear. Fourth, long-term outcomes in infants exposed to maternal SARS-CoV-2 infection—including neurodevelopmental, immunological, and metabolic trajectories—require systematic longitudinal evaluation. Finally, optimal vaccination timing and booster strategies for maximizing neonatal protection warrant further investigation.

### 8.7. Future Directions

Future research should prioritize integrative, systems-level approaches that combine multi-omics, spatial transcriptomics, proteomics, and advanced computational analyses to clarify how maternal inflammation intersects with placental microenvironments throughout gestation [83]. Variant-adapted vaccine platforms should be evaluated for durability, cross-variant protection, and efficiency of transplacental antibody transfer. Predictive machine learning models leveraging clinical and molecular data may improve early risk stratification for placental dysfunction, preterm birth, and hypertensive disorders. Additionally, targeted therapies addressing endothelial injury, complement activation, and angiogenic imbalance represent promising avenues for reducing maternal and fetal morbidity. Expansion of global pregnancy registries will be essential to capture variant-specific outcomes, booster effectiveness, and treatment safety profiles at scale.

An additional consideration is the potential role of intra-host viral evolution. As an RNA virus, SARS-CoV-2 exists as a dynamic quasi-species population, comprising genetically related variants that may emerge during replication within an individual host. In prolonged or severe infections, selective pressures from immune responses could theoretically favour variants with altered receptor affinity or immune evasion capacity. While direct evidence linking intra-host viral diversification to altered placental tropism remains limited, this evolutionary plasticity warrants further investigation, particularly in the context of pregnancy where immune modulation may influence viral persistence and adaptation.

## 9. Conclusions

SARS-CoV-2 infection during pregnancy represents a complex, multisystem disorder in which adverse maternal and fetal outcomes arise predominantly from immune-mediated and vascular mechanisms rather than direct placental viral infection. Accumulating clinical, molecular, and placental evidence indicates that maternal systemic inflammation, endothelial dysfunction, thrombo-inflammatory signaling, and angiogenic imbalance collectively impair placental perfusion, trophoblast function, and endocrine stability, thereby increasing the risk of hypertensive disorders, fetal growth restriction, and preterm birth.

The rarity of vertical transmission underscores the limited biological permissiveness of the placenta to productive viral replication; however, the maternal–fetal unit remains highly sensitive to inflammatory and vascular stressors triggered by SARS-CoV-2. Preventive strategies, particularly vaccination, play a central role in mitigating these pathological cascades by reducing disease severity, preserving placental vascular integrity, and providing effective passive immunity to the neonate. Adjunctive therapeutic interventions, including antiviral agents, anticoagulation, and corticosteroids, further contribute to improved outcomes when applied in accordance with evolving clinical guidelines.

As SARS-CoV-2 continues to evolve, future progress will depend on integrated research frameworks that combine molecular profiling, placental biology, clinical phenotyping, and large-scale pregnancy registries. Such approaches are essential for refining risk stratification, optimizing vaccination and treatment strategies, and ultimately improving maternal and neonatal health outcomes in the context of ongoing and future viral threats.

## Data Availability

No new data was created.

## Abbreviations

ACE2	Angiotensin-converting enzyme 2
ACOG	American College of Obstetricians and Gynecologists
COVID-19	Coronavirus disease 2019
CTSL	Cathepsin L
FURIN	Proprotein convertase subtilisin/kexin type 3
IFN	Interferon
IRB	Institutional Review Board
NRP1	Neuropilin-1
RBD	Receptor-binding domain
SARS-CoV-2	severe acute respiratory syndrome coronavirus 2
TMPRSS2	Transmembrane serine protease 2
WHO	World Health Organization
